# Analysis of Local Properties and Performance of Bilayer Epitaxial Graphene Field Effect Transistors on SiC

**DOI:** 10.3390/ma17143553

**Published:** 2024-07-18

**Authors:** Dalal Fadil, Wlodek Strupinski, Emiliano Pallecchi, Henri Happy

**Affiliations:** 1 University of Lille—IEMN CNRS UMR 8520, Avenue Poincaré, CS 60069, 59652 Villeneuve d’Ascq, France; emiliano.pallecchi@univ-lille.fr; 2 Departament d’Enginyeria Electrònica, Universitat Rovira I Virgili, 43007 Tarragona, Spain; 3 Faculty of Physics, Warsaw University of Technology, Koszykowa 75 Str., 00-662 Warsaw, Poland; wlodek.strupinski@pw.edu.pl

**Keywords:** bilayer graphene, silicon carbide, field effect transistors, nanofabrication, Raman spectra analysis, DC and RF characterizations

## Abstract

Epitaxial bilayer graphene, grown by chemical vapor deposition on SiC substrates without silicon sublimation, is crucial material for graphene field effect transistors (GFETs). Rigorous characterization methods, such as atomic force microscopy and Raman spectroscopy, confirm the exceptional quality of this graphene. Post-nanofabrication, extensive evaluation of DC and high-frequency properties enable the extraction of critical parameters such as the current gain (*f_max_*) and cut-off frequency (*f_t_*) of hundred transistors. The Raman spectra analysis provides insights into material property, which correlate with Hall mobilities, carrier densities, contact resistance and sheet resistance and highlights graphene’s intrinsic properties. The GFETs’ performance displays dispersion, as confirmed through the characterization of multiple transistors. Since the Raman analysis shows relatively homogeneous surface, the variation in Hall mobility, carrier densities and contact resistance cross the wafer suggest that the dispersion of GFET transistor’s performance could be related to the process of fabrication. Such insights are especially critical in integrated circuits, where consistent transistor performance is vital due to the presence of circuit elements like inductance, capacitance and coplanar waveguides often distributed across the same wafer.

## 1. Introduction

Graphene is considered one of the most famous two-dimensional (2D) materials of this century [1,2,3,4]. This honeycomb carbon atom lattice exhibits extraordinary electrical [5,6], optical [7], thermal [8] and mechanical [9] properties and attracts huge attention for a wide panel of device applications, especially for radio-frequency transistors on rigid or on flexible substrate [4,10,11,12]. Since 2004, researchers continue improving the technique of growth, either by exfoliation [13,14,15] or by chemical vapor deposition (CVD). The most common technique used to grow graphene is using CVD on copper and transferring the carbon monolayer to a host substrate [16,17]. However, efforts remain to be made regarding the reliability and the reproducibility of the quality after transfer due to the cracks, wrinkles and residues, as reported by Smith et al. [18]. Another alternative is to grow graphene by CVD directly on SiC substrate by graphitization or by epitaxial growth without SiC sublimation or by hydrogen intercalation [19,20,21,22,23,24,25]. Besides the large-scale fabrication, the main advantage of CVD on SiC is to avoid the transfer from copper and improve the electronic properties of the material by using hydrogen intercalation, as reported by Ciuk et al. [26,27]. One of the advantages of the bilayer graphene on SiC substrate is overcoming the lack of a band gap; other possibilities reported in the literature include introducing defects, doping, strain and chemical bounding to the substrate [28,29,30]. Furthermore, the bilayer graphene field effect transistors (GFETs) on SiC have demonstrated better performances than monolayer GFETs [31]. The 100 nm gate length (*L_g_*) and 2 × 4 µm channel length (*W_g_*) transistor exhibits 60 GHz of extrinsic current gain cut-off frequency *f_T_extr_* and 25 GHz of maximum frequency of oscillation *f_max_extr_*, and by annealing the bilayer graphene in hydrogen, the performance can be enhanced and reach *f_T_extr_* = 70 GHz and *f_max_extr_* = 120 GHz for *L_g_* = 60 nm and *W_g_* = 2 × 8 µm [32]. To analyze graphene properties, the standard non-destructive techniques used are (i) the atomic force microscopy AFM that give information about surface morphology including defects like holes, wrinkles or grain boundaries (ii) Raman spectroscopy which provides information about the number of layers, doping, strain and disorders in the graphene surface [33,34]. A new specific technique is non-contact Terahertz time domain spectroscopy. This complementary technique gives large scale information about mobility and carrier density [35].

The motivation behind this study goes beyond evaluating the RF performance of a single transistor. It also emphasizes the reproducibility of transistor performance across a large area, investigating whether the growth was uniform and the process reproducible. Understanding the material’s properties and analyzing the local properties, mobility, contact resistance and their impact on device performance is crucial for enhancing the reliability and uniformity of transistors in integrated circuits.

In this work, we report the fabrication process of bilayer graphene field effect transistors. Initially, we verify the quality of the graphene samples before fabrication using AFM and Raman spectroscopy. Post-fabrication, we conduct further analysis using Raman spectroscopy, Hall mobility and TLM measurements to assess the local properties and the homogeneity of the samples. To evaluate device performance across the entire wafer, we performed DC and RF characterizations on hundreds of transistors. The RF performance data were then compared with the local properties of the samples, supporting a conclusion about device variability across the wafer. Our findings demonstrate significant performance variation across the wafer. The average <*f_t_*>/<*f_max_*> ranged from 3/0.5 GHz in cell 4 to 16/11 GHz in cell 7 for devices with *L_g_* = 200 nm and *W_g_* = 2 × 30 µm. Notably, the devices in cell 7 and 8, which exhibited the best performance, also had the lowest contact resistance. This work highlights the important role of the fabrication process in the RF performance variability of graphene FETs fabricated from high-quality epitaxial bilayer graphene on a SiC substrate.

## 2. Materials and Methods

Epitaxial bilayer graphene was synthesized by chemical vapor deposition on a 500 µm thick, high-resistivity 6H-SiC (0001) substrate using a commercial horizontal CVD hot wall Aixtron VP508 reactor (Aixtron, Herzogenrath, Germany) equipped with an RF generator for heating. Prior to growth, in situ etching of the SiC surface was performed under a hydrogen atmosphere at 1600 °C and a chamber pressure of 100 mbar. The carbon films were deposited using propane as the carbon precursor. Our method employs high-temperature and low-argon-pressure CVD, creating laminar argon flow dynamics to protect the SiC substrate from silicon sublimation and facilitate propane mass transport, thereby enabling graphene epitaxy, as detailed in references [22,25,26]. The growth process was followed by in situ hydrogen intercalation at 1000 °C in a 900 mbar Ar atmosphere. The optimization of growth parameters aimed at achieving uniform bilayer graphene. Before fabrication, initial carrier density and the mobility was provided by Ciuk et al. to be around +8.3 × 10^12^ cm^−2^ and 850 cm^2^·V^−1^s^−1^, respectively. In contrast to the methodology described by P. Wehrfritz et al. [36], which utilized a similar SiC substrate, our process involves unique conditions including high-temperature in situ hydrogen etching and controlled low-argon pressure to prevent silicon sublimation. These optimizations contribute to the superior uniformity and controlled thickness of our bilayer graphene compared to prior studies. Notably, our approach introduces an innovative in situ hydrogen intercalation step at 1000 C, enhancing graphene quality, which was not emphasized in previous literature [36].

The AFM Bruken Icon model was used on mode tapping to determine the properties, the material and image of the surface. Image analysis was performed with WSxM5.0 Develop 8.3, a free software. High-resolution electron beam lithography VISTEC EBPG5000Plus was used for device fabrication. To analyze the local properties of the material, we used a HORIBA Jobin–Yvon lab system for Raman spectroscopy at a laser wavelength of 473 nm, using a 1 µm laser spot size and filters to deliver power less than 0.1 mW and ×100 objective lens to measure the different positions of the sample. HL5500PC was used to carry out Hall measurement. To measure the DC and RF performance of hundreds of devices, we used a standard probe station with Microtech’s probes, Semiconductor Analyzer HP4155A, the Vector Network Analyzer HP4155A and the vector Network Analyzer Rohde & Schwarz ZVA67.

A. Before fabrication

**Figure 1:**
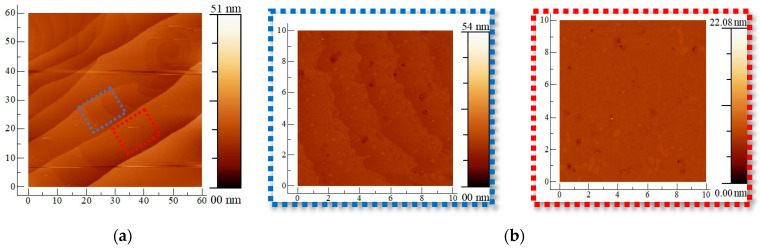

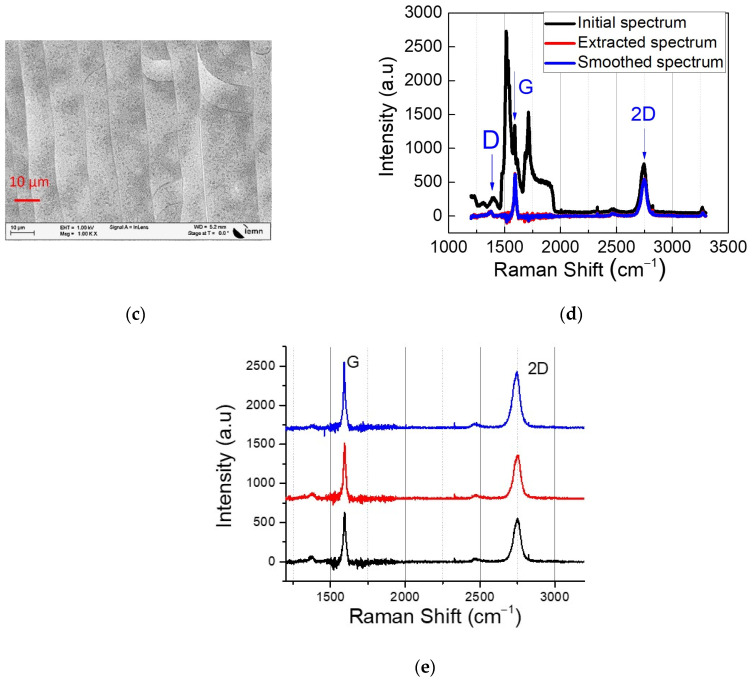
AFM images of bilayer graphene surface on 6H-SiC substrate [40]. (**a**) 60 × 60 µm^2^ image and the blue and red dash square 10 × 10 µm^2^ is represented in (**b**). (**c**) SEM image with 10 µm scale bare. (**d**) Raman spectroscopy of graphene on SiC substrate. The black trace is the spectrum of graphene and SiC, the red trace is the graphene spectrum once SiC Raman peaks are subtracted, the blue trace is the smoothed spectrum. (**e**) Raman spectra at different locations (blue, red, and black) on the SiC wafer.

Primary characterization was performed before fabrication. Figure 1a represents atomic force microscopy (AFM) images of a 60 × 60 µm^2^ surface area of graphene observed at room temperature. The AFM images reveal well-oriented, parallel atomic steps with SiC terraces approximately 10 µm wide, separated by steps estimated to be a few nanometers in height. This phenomenon, known as step bunching, occurs during the growth-preceding in situ hydrogen etching of the SiC surface, as described in reference [27]. Within these terraces, Figure 1b showcases two distinct surface morphologies. The graphene roughness within individual terraces has a root mean square (RMS) value of 0.273 nm (Figure 1b, left) and 0.2406 nm (Figure 1b, right), significantly smoother than the 1 nm roughness reported in previous studies [36]. Figure 1c shows the SEM image of epitaxial graphene on the SiC substrate. We observed the graphene surface nucleation on the SiC steps. An example of a Raman spectroscopy of graphene on SiC and after extracting SiC peaks is presented in Figure 1d. We observed small intensity of the D peak compared to the G and 2D peaks, indicating a small disorder and defects in the material. Previous work about Raman studies in graphene on SiO_2_/Si show that the shape of the 2D peak is an indicator of the number of layers [37]. For Raman analysis, the nature of the growth (exfoliation, CVD graphitization, CVD without graphitization) and the type of the substrates (SiO_2_ or SiC) can affect the peaks’ position and the value of the full width at half maximum (FWHM), which enhance the need to have more results about bilayer graphene on SiC [26,38,39]. Here, the full width at the half maximum of the 2D peak is around 59 cm^−1^, comparable to the 41–62 cm^−1^ reported in bilayer graphene on SiC and other substrates [25,37]. In bilayer graphene, the 2D peak is typically broader and upshifted compared to monolayer graphene. This broadening is due to the presence of an additional phonon mode in bilayer graphene, resulting in a more complex 2D band shape [37]. Before fabrication, Raman spectra was established in three different locations randomly selected on the SiC wafer and presented in Figure 1e. The Raman spectrum does not change with changing locations.

B. Device fabrication.

**Figure 2:**
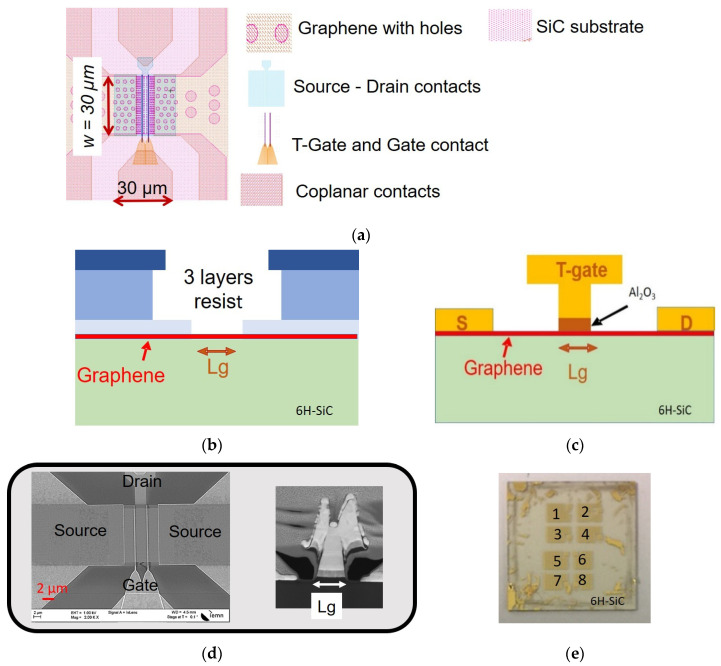
(**a**) A picture of the layout of the device. “+” is source-drain contact layer. (**b**) A schematic image representing the step of the realization of the T gate process with the three layers’ resists. (**c**) A schematic of the side view of the T gate transistor. (**d**) (**d**, **left**) An SEM image of the transistor in the end of the process. (**d**, **right**) An FIB image of the transistor showing the T-gate structure, *L_g_* = 200 nm. (**e**) A photograph of the final devices fabricated on the 15 mm × 15 mm SiC substrate, including 458 transistors and height cells numbered from 1 to 8.

The field effect transistors based on graphene were fabricated on a 15 × 15 mm^2^ SiC wafer. Figure 2a represents the layout of the device. Each level of the layout is represented by a different color or contrast and represents a fabrication step of the process. First, the process is fixed by defining the alignment marks. It follows the etching of the graphene channel and contacts as a hole for improving contact resistance, as previously reported in [41,42]. The source and drain contacts were obtained by the standard lift-off process after evaporating 1.5 nm of nickel and 30 nm of gold metals. Here, a thin layer of nickel (1.5 nm) was deposited before in order to improve the metal adhesion on the surface. The dual T-gate with gate length (*L_g_*) were defined by using three layers of poly-meta-methacrylate (PMMA), as shown in Figure 2b, where three different thicknesses were defined: 160 nm thick at the bottom part of the T-gate, 720 nm thick at the top part of the T-gate and 130 nm at the resist followed by electron beam (e-beam) lithography. After the development of these multilayers’ resists, the gate oxide is deposited using 2 nm of evaporated aluminum four times, followed by oxidation in ambient air for 24 h. Finally, the coplanar access Ni (50 nm)/Au (300 nm) are deposited, followed by lift-off. The cross-section schema of the final transistor is presented in Figure 2c. The top view of the active part of the final dual-T-gate-transistor is illustrated in the scanning electron microscopy (SEM) image in Figure 2d (left) while the cross-section of the gate part was illustrated by the focused ion beam (FIB) technique in Figure 2d (right) and shows the shape of the final T-gate of the GFET. A picture of the 15 × 15 mm^2^ final wafer where there are 8 cells and 458 transistors is shown in Figure 2e.

## 3. Results and Discussion

**Figure 3:**
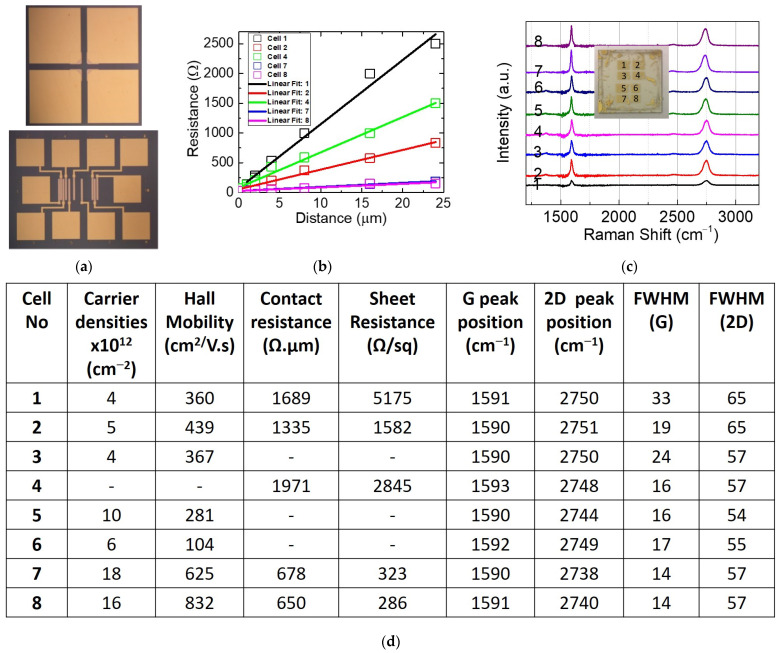
(**a**) An optical image of the Hall and TLM pattern. (**b**) The transmission line measurement (TLM) and the linear fit to extract the correspondent contact resistance and sheet resistance. (**c**) A recapitulation of the averaged Raman spectra of graphene on SiC measured in the eight cells of the wafer. (**d**) A table summarizing the values of the Hall mobility, carrier densities and Raman peaks’ positions and the full width at the half maximum of the G and 2D peaks. Missing values are due to faulty components.

After fabrication, we conducted an in-depth characterization of the electrical properties of graphene. The wafer was tested within patterned structures, including Hall measurements and transmission line measurement (TLM), as illustrated in Figure 3a, across various cells. Contact and sheet resistance, which represent the resistance as a function of the distance between contact points (0.5, 1, 2, 4, 16, 24 µm), were extracted from a linear fit using the TLM method. The *y*-axis intercept provides the contact resistance (2Rc), while the slope of the linear fit represents the Rsh ratio to the width of the graphene channel [41,43]. All extracted values of contact resistance and sheet resistance for each cell are presented in Figure 3d.

To complete the analysis, we acquired seven Raman spectra from each cell of the wafer and extracted an average Raman spectrum. Figure 3c summarizes the average Raman spectra representing each cell. Mobility values, carrier densities and Raman peaks are provided in the table in Figure 3d. It shows a dispersion of values for contact resistance, sheet resistance, Hall mobility and carrier density across the wafer. The highest Hall mobility (626–832 cm^2^/V·s), carrier densities 18–16 (×10^12^ cm^−2^), lowest values of contact resistance (678–650 Ω·m) and sheet resistance (323–286 Ω/sq) were obtained in the cells 7–8. However, the low value of mobility observed in cells 1–6 is possibly due to the high contact resistance. The ohmic contact between graphene and metal contacts exhibits large contact resistance, significantly reducing the apparent mobility of contacted graphene and hindering its potential in high-frequency applications [42].

From the Raman analysis, the position of the G peak remains consistent across the wafer, while the full width at the half maximum of the G peak FWHM (G) varies. The thinnest FWHM (G) is observed in cells 7 and 8. The 2D peak exhibits a blueshift of approximately 20 cm^−1^, from 2751 cm^−1^ in cell 2 to 2731 cm^−1^ in cell 7. This shift could be attributed to interlayer coupling effects in bilayer graphene. Ciuk et al. [25] investigated bilayer CVD graphene on SiC and observed the blueshift of the 2D peak and redshift of the G peak due to the strain at the edges of the steps. Moreover, Das et al. [44] demonstrated that in graphene on SiO_2_/Si, the position of the G peak increased, while the 2D peak and the FWHM (G) decreased with increased dopant concentration. Ferrari et al. [37] showed that the electron and hole doping upshifts and sharpens the G peak. Additionally, graphene can be doped chemically during fabrication, affecting carrier densities, 2D-peak width and position. Previous studies have shown that doping increases the 2D position due to the modification of lattice parameters, which modifies the total number of charges and leads to a stiffening/softening of the phonons [45]. The analysis of the table in Figure 3d clearly shows that cells 7–8 present the highest mobility, carrier densities, lowest contact and sheet resistance and smallest FWHM (G), suggesting favorable prospects for effective device and circuit applications.

To evaluate the performance of our devices, DC and RF measurements were performed using a standard probe station with Microtech’s probes, Semiconductor Analyzer HP4155A, the Vector Network Analyzer HP4155A and the vector Network Analyzer Rohde & Schwarz ZVA67. A common calibration procedure of Line-Reflect-Reflect-Match (LRRM) for RF measurements was established before measurements. In total, 195 transistors of the GFETs were measured.

**Figure 4:**
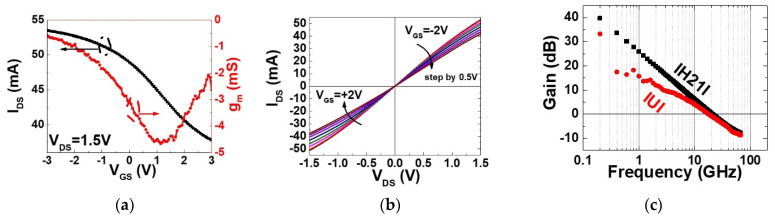
Example of DC and RF characteristics of dual T-gate graphene transistor in cell 7 having gate channel length (*L_g_*) equal to 200 nm and dual-gate width (*w*) 2 × 30 µm. (**a**) DC measurement *I_DS_*–*V_GS_* and *g_m_*–*V_GS_*. (**b**) The voltage transfer characteristics as a function of *V_GS_* varying from −2 V to +2 V by 0.5 V steps. (**c**) RF characteristic includes the as-measured values of the current gain *H_21_* and the unilateral power gain *U* as a function of the frequency at *V_DS_* = 1.5 V and *V_GS_* = 1.3 V. The cut off frequency *f_t_* and the maximum oscillation frequency *f_max_* have been extracted [40].

Figure 4 represents an example of the electrical characterization of a transistor in cell 7 (Figure 2e), with a 200 nm gate length and a 30 × 2 µm channel width. The transfer characteristic *I_DS_* as function of *V_GS_* at *V_DS_* = 1.5 V and the transconductance (*g_m_* = d*I_DS_*/d*V_GS_*) are shown in Figure 4a. The *I_DS_*–*V_GS_* curve is non-monotonic. As *V_GS_* changes from −3 V to +3 V, *I_DS_* shows a change in slopes initially decreasing, then reversing direction. This non-monotonic behavior is accurately reflected in the *g_m_* curve, calculated as the derivative of *I_DS_* as a function of *V_GS_*_._ The characteristic of decreasing and then increasing *g_m_* is consistent with our observations and not indicative of bipolar behavior as observed in [46]. The on/off extracted is approximately 1.4, within the *V_GS_* range of ±3 V. The peak *g_m_* reaches 4.6 mS (76 mS/mm) at *V_GS_* = 1.3 V. The Dirac point is located at *V_GS_* higher than 3 V, suggesting p-type doping in the graphene channel. Figure 4b shows the output characteristics, sweeping the gate voltage from −2 V to +2 V in 0.5 V steps. The maximum current is around 53 mA (0.88 mA/µm) at *V_DS_* = +1.5 V and *V_GS_* = −2 V, with no observed saturation current. This work investigates graphene’s potential for high-frequency analog applications, focusing on its high-frequency characteristics rather than switching capability and looking for it to reach the saturation current. Graphene’s semi-metallic nature results in a low on/off ratio, which is less critical for RF amplifiers compared to synaptic transistors or for digital applications [12,46]. Due to its high carrier mobility and conductivity, graphene excels in GHz frequency operations, maintaining steady current mobility flow at specific bias points [47].

The extrinsic RF characterization is reported in Figure 4c. It shows the current gain H_21_ and the Mason’s gain U of the device in a frequency range of 0.6 to 67 GHz. The maximum frequency of oscillation (*f_max_*) and the current gain cut-off frequency (*f_t_*) are, respectively, the frequency at which the power gain (*U)* and the current gain (*H*_21_) are equal to 1. The on-probe measurement of the cut-off frequency and the maximum oscillation frequency reveal, respectively, *f_t_* = 21 GHz and *f_max_* = 18 GHz at *V_GS_* = +1 V and the gain curves follow the slope of 20 dB/decade, as expected. The extrinsic value of the performance is obtained after the capacitances related to the length of the transmission line of transistor access are removed [48]. They are, respectively, *f_T-extr_* = 62 GHz and *f_max-extr_* remains the same.

These values are comparable to the recent value achieved by [32] in the bilayer graphene on SiC.

**Figure 5:**
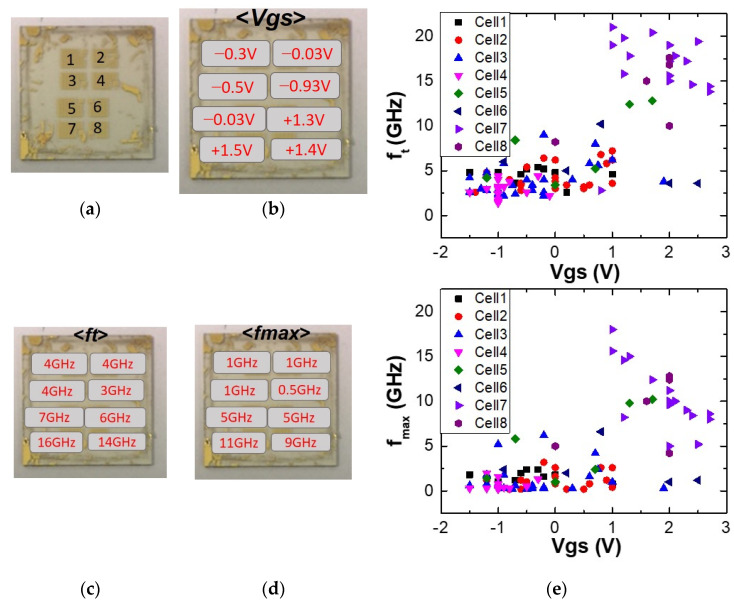
(**a**) Photography of the 15 mm × 15 mm graphene wafer presented previously in Figure 2e. (**b**) *V_GS_* is the bias gate voltage related the maximum the transconductance and where the best performance of the transistor is expected. <*V_GS_*> is the average value of all the gate voltage measured in each cell. (**c**,**d**) Representation of the average values of the cut-off frequency <*f_t_*> and the maximum oscillation frequency <*f_max_*> computed for each cell. (**e**) Graph summarizing the evolution of the on-probe values of *f_t_* and *f_max_* as function of the biased gate voltage *V_GS_* of the transistors in each cell.

To understand the evolution of RF performance across the eight cells of the full wafer shown in Figure 5a, we analyzed the average values of *f_t_*, *f_max_* and the optimum gate voltage for each individual cell. The optimum gate voltages for RF measurement were determined from the *g_m_*—*V_GS_* characteristic, where the transconductance is at its maximum. The average gate voltage <*V_GS_*> is shown in Figure 5b and represents the mean gate voltage computed in each cell. The average cut-off frequency <*f_t_*> and maximum oscillation frequency <*f_max_*> are reported in Figure 5c,d, respectively. They clearly show that the best performance is achieved in cells 7 and 8, with <*f_t_*> of 16–14 GHz and <*f_max_*> of 11–9 GHz, respectively. Figure 5e summarizes the *f_t_* and *f_max_* values for each cell as a function of the optimum gate voltage. Interestingly, the best transistor performances are obtained in cells 7 and 8 for gate voltages of at least 1 V. This variation in gate voltage may be attributed to the variation of the dopant across the wafer as mentioned in the Raman analysis. Additionally, differences in contact resistance across various cells contribute to the observed variability. To mitigate this issue, implementing a robust cleaning process is essential to eliminate polymer contamination during fabrication. This approach will reduce contact resistance, enhance uniformity, and minimize performance variability. It is important to acknowledge that both contact resistance and graphene quality are important for overall device performance. However, our study suggests that the RF performance is immediately limited by contact resistance related to the process of fabrication.

The findings highlight the critical role of the fabrication process in the RF performance variability of graphene FETs fabricated from high-quality epitaxial bilayer graphene on a SiC substrate. Despite the material’s exceptional quality, confirmed through AFM and Raman spectroscopy, the observed dispersion in values of Hall mobility, carrier densities and contact resistance across the wafer suggest that these performance discrepancies result from the fabrication stages rather than inherent material inconsistencies. This understanding underscores the need for refined manufacturing techniques to ensure consistent transistor behavior, which is essential for the reliable operation of integrated circuits that rely on uniform performance of GFET in wafer scale. The insights gained from this study are essential for advancing GFET technology, particularly in high-frequency applications where reproducible device characteristics are crucial.

## 4. Conclusions

In this paper, we fabricated hundreds of bilayer graphene field effect transistors on a SiC substrate. We provided Raman spectrum analysis combined with the Hall mobility, carrier densities, contact resistance and sheet resistance toward a 15 mm × 15 mm graphene wafer. The analyses of local Raman have shown that the 2D peak and the FWHM (G) changed, as well as the Hall mobility, carrier densities and contact resistance in the wafer. The analysis of the RF performances was performed and compared to the local properties of the wafer. It revealed dispersion of the performances and correlation between the RF performance, contact resistance and Hall mobilities. This work completes the few works about local Raman investigation on bilayer graphene on SiC and highlights the importance of local analyses of the properties of the material to evaluate the performance of electronic devices where large-scale, homogenous and high-quality fabrication of the material is needed for high-performance circuit.

## Data Availability

Data is contained within the article.
